# Electrophysiological trajectories of concussion recovery: From acute to prolonged stages in late teenagers

**DOI:** 10.3233/PRM-210114

**Published:** 2023-06-07

**Authors:** Mo Mortazavi, Francesca Arese Lucini, David Joffe, David S. Oakley

**Affiliations:** a SPARCC Sports Medicine, Rehabilitation, and Concussion Center, Tucson, AZ, USA; b Department of Pediatrics, Tucson Medical Center, Tucson, AZ, USA; c WAVi Research, Boulder, CO, USA

**Keywords:** Acute concussion, prolonged post-concussion syndrome, return to play, electroencephalogram, event related potentials

## Abstract

**PURPOSE:**

Numerous studies have reported electrophysiological differences between concussed and non-concussed groups, but few studies have systematically explored recovery trajectories from acute concussion to symptom recovery and the transition from acute concussion to prolonged phases. Questions remain about recovery prognosis and the extent to which symptom resolution coincides with injury resolution. This study therefore investigated the electrophysiological differences in recoveries between simple and complex concussion.

**METHODS:**

Student athletes with acute concussion from a previous study (19(2) years old) were tracked from pre-injury baseline, 24–48 hours after concussion, and through in-season recovery. The electroencephalography (EEG) with P300 evoked response trajectories from this acute study were compared to an age-matched population of 71 patients (18(2) years old) with prolonged post-concussive symptoms (PPCS), 61 (SD 31) days after concussion.

**RESULTS:**

Acute, return-to-play, and PPCS groups all experienced a significant deficit in P300 amplitude compared to the pre-injury baseline group. The PPCS group, however, had significantly different EEG spectral and coherence patterns from every other group.

**CONCLUSION:**

These data suggest that while the evoked response potentials deficits of simple concussion may persist in more prolonged stages, there are certain EEG measures unique to PPCS. These metrics are readily accessible to clinicians and may provide useful parameters to help predict trajectories, characterize injury (phenotype), and track the course of injury.

## 1 Introduction

Concussion is a brain injury that can lead to cognitive, physical, and/or psychological impairment. Concussions are difficult to identify, and to date, there is no objective protocol or marker that is specific for diagnosis [1–3]. Most mild traumatic brain injuries (mTBIs) last for approximately one month, but around 10–30% of the injuries can persist beyond that timeframe, giving rise to persistent post-concussion syndrome (PPCS), a condition characterized by strong headaches, dizziness, fatigue, loss of concentration, etc [4–9]. Today, clinicians rely heavily on neuropsychological (NP) assessments to help in the evaluation of brain trauma as these assessments provide insight on the functional integrity of the brain and the levels of cognitive impairment and can therefore help in treatment and return-to-play decisions [10–13].

Electroencephalography (EEG) and EEG with auditory or visual evoked response potentials (ERPs) have been studied as potential tools to aid in the diagnosis of acute and/or prolonged concussion alongside NP assessments [14–40]. EEG is the recording of the electrical signal generated by the brain through electrodes placed on the scalp, and ERPs are measurements of the EEG signal time-locked to the onset of visual or auditory stimuli [41–47]. Studies have shown both the amplitude and delay (latency) of these evoked responses to be sensitive to various conditions accompanied by cognitive impairment [45–47]. Numerous studies have reported ERP-related differences between mTBI and control groups, even as some of these found no differences using NP measures [30–40]. Clinicians and scientists have studied EEG and ERPs for mTBI and PPCS because of the relative ease, objectivity, and sensitivity these tests can provide, ultimately hoping they can help determine optimal management strategies.

The general goal of this study was to analyze electrophysical changes and understand the trajectory of EEG-related biomarkers after a concussive event over time by comparing these markers in simple and complex concussion cases and against baseline groups. Following the lead of previously published work, three different sets of EEG measures were investigated to assess cerebral activity in these groups.

The first set of measures involved an auditory odd-ball P300. This is a common ERP protocol that measures the brain’s response to an odd or rare tone which typically presents as a positive EEG voltage occurring roughly 300 ms after the delivery of the odd-ball stimulus. The amplitude of the P300 response is considered to be proportional to the number of attentional resources devoted to detecting the rare tone where larger amplitudes and smaller latencies (faster processing speeds) have been associated with superior information processing [41–49]. The P300 amplitude has been seen to decline after a concussion with deficits sometimes lingering after symptom resolution [29–33, 40].

The second set involved spectral analysis, which characterizes the frequency composition of EEG, where peak frequencies and power have been reported to notably change after trauma [14–22]. In the acute concussion phase, a reduction in peak alpha frequency is commonly reported after mTBI, but the frequency of the posterior alpha rhythm may increase in the weeks to months after mTBI, which has been posited to be a return to the original baseline from the post-traumatic slowing [19]. As with P300, these changes often linger in the acute phase even after symptoms or NP deficits have resolved [21]. In PPCS subjects, electrophysical abnormalities such as lower amplitudes in the ∼8–12 Hz frequency band (alpha) have been reported as lasting for years in persistent PPCS but as reversable in transient PPCS [16–20].

Reduced alpha amplitudes have been associated with diminished cognitive function where the reduced posterior cortical alpha activity has been hypothesized to be related to injuries in the neocortex region [16–21].

The third set involved coherence, which correlates EEG between two channels to assess how similar or “coherent” the underlying brain activity is as a function of frequency. Coherence has been shown to increase following mTBI, particularly in the long interhemispheric connections, and some of these abnormalities seem to persist after symptom resolution or after a return to normal performance on NP evaluations [21–27].

It has been posited that concussed participants may recruit additional brain resources to mask their inability to produce the necessary level of amplitude, implying that functional resolution happens faster than physiological resolution [21]. Current clinical concussion assessment tools may not be sensitive enough to detect these subtle concussive deficits.

## 2 Methods

P300 amplitudes and latencies, spectral information (alpha frequency and amplitude), and coherence measures for PPCS patients were compared to subjects in a simple-concussion group. The simple-concussion group was comprised of athletes (aged 17 to 23, 19(2) years) participating in contact sports; they were measured at pre-contact baseline, after concussion (acute), within-season return to play, and at the end of the season. These athletes did not transition to a chronic phase (or PPCS) and returned to their respective sports. In order to compare acute to prolonged concussion, a retrospective cross-sectional analysis of PPCS cases was also done on a different group of patients aged 17 to 23 (18(2) years) with persistent post-concussive symptoms and functional neurologic deficits lasting longer than one month after injury. Both cohorts were compared to age-matched baseline testing of the acute group before injury as well as non-injured athletes using the same protocol (and hardware) from a multitude of sites.

## 2.1 Subjects

The acute concussion data and the control group for this study were from a previous study that followed athletes over the course of up to four sports seasons and at five different sites [32]. The injury groups were compared to the pre-injury baselines (to get a sense of trajectory), and a reference group was taken from pre-season baselines of 120 subjects who were not part of the acute concussion group and who had no history of previous concussion.

The concussed subjects were tested, when practical, at pre-contact baseline, 24–48 hours after a concussive event, during graded return-to-play protocols (presumably symptom-free), and at post-season several months removed from the event. Return to play for the concussion group was guided by the attending physician, utilizing an assessment and management system from the International Symposia on Concussion in Sport to evaluate return to play after concussion [1].

PPCS subject data were collected on 71 patients, average 18(2) years of age, from a concussion clinic specializing in treating prolonged post-concussion symptoms. Brain injury was sustained from a variety of different mechanisms including sports injury, falls, and motor vehicle accidents. Inclusion criteria were prolonged symptoms defined as > 30 days and moderate to severe clinical symptoms based on the Sport Concussion Assessment Tool 5th Edition (SCAT5) and clinical evaluations. All patients with PPCS had moderate to severe clinical symptoms with limited academic and exertional tolerance. Cognitive testing (impact), vestibular testing (King-Devick [KD], Near Point of Convergence [NPC], Bertec force plate, Vestibular Ocular Motor Screening [VOMS]), and monitored exercise testing (Buffalo Concussion Treadmill Test [BCTT] and Buffalo Concussion Bike Test [BCBT]) were completed in all patients to objectively assess functional deficits. Patients were excluded if they had a history of learning disorders, seizure disorder, or complex concussions with a skull fracture or intracranial hemorrhage.

Table 1 summarizes the number of tests performed for each category after low yield removal as explained below. The study was approved by the Solutions Institutional Review Board, and written informed consent was obtained from the participants or their parents before study intake.

**Table 1:** Assessments and mean timing of assessments

*Group*	# *of Subjects*	*Timing of Assessments (*±*SD)*
Reference (REF)	120	–
*(assessments of non-injured players)*	(91 male, 29 female)
Simple Concussion:
Pre-injury baseline (BASE)	33	–
*(initial assessments of concussion group before injury)*	(27 male, 6 female)
Acute concussion (Acute CN)	33	1.6 (1.2) days after event
*(assessments of concussion group after concussion event)*	(27 male, 6 female)
Acute Return-to-play (Acute RTP)	32	10 (5) days
*(concussion group at beginning of return-to-play protocols, presumably symptom-free)*	(26 male, 6 female)	after event
Acute post-season (Acute CEND)	24	94 (35) days
*(concussion group at post-season)*	(18 male, 6 female)	after event
Complex concussion (PPCS)	63	61 (31) days
*(assessments of PPCS patients on initial PPCS visit)*	(42 female, 21 male)	after event

## 2.2 EEG acquisition and preprocessing

**Fig. 1:**
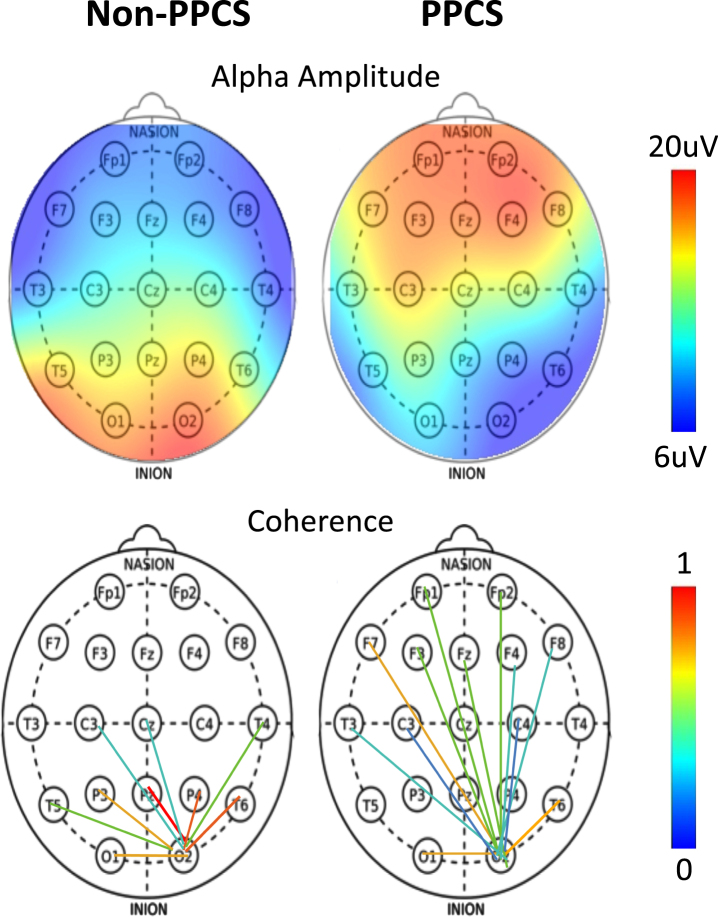
(Top) Alpha amplitude as a function of scalp location for typical non-PPCS and PPCS patients. (Bottom) Occipital connectivity from O2 electrode as a function of scalp location for typical non-PPCS and PPCS patients.

The EEG was recorded using the WAVi ® Research Platform (WAVi Research, Boulder, CO, USA) sampled at 250 Hz and bandpass filtered between 0.5–30 Hz. The electrodes were placed according to the International 10–20 system (Fig. 1), and linked reference electrodes were placed at the earlobes. Test administrators were instructed to establish electrode impedances below 30 k*Ω* for EEG locations and below 20 k*Ω* for the ground-to-ear locations where possible. These targets were well below the 1 G*Ω* input impedance of the WAVi amplifiers, were practical regarding preparation time, and produced sufficient yield [50].

The testing details for the PPCS group were the same as those previously described, with a continuous four-minute, two-tone, eyes-closed protocol presenting 200 common tones (1000 Hz) and 40 rare tones (2777 Hz) in random order once per second [32]. This protocol was used to acquire both background EEG and evoked ERP data as discussed below.

## 2.3 EEG extraction

EEG was extracted using WAVi Desktop software (version 0.9.8.18), in which spectral information was extracting using standard Fourier transform methods. All trials included automatic artifact rejection, which excluded sections of EEG data with higher than acceptable amplitudes and excessive band frequency activities in the standard EEG bands (delta, theta, alpha, and beta) on an individual channel basis. Files were also manually inspected to confirm proper artifact detection. Segments of data that contained synchronized eye blinks were also excluded from the study.

## 2.4 EEG measurements

### 2.4.1 ERP

The depth (P300 V) was extracted from the mean amplitude of the response to all stimuli. The amplitudes of the P300 components reported here were measured by identifying the average positive extremum in the latency range of 240–500 ms relative to the average of the first 16 ms post-stimulus, baseline corrected using the 100 ms pre-stimulus period. To be consistent with the previous study of acute concussion, [32] the highest P300μV from the 6 C-P scalp sites (C3, CZ, C4, P3, Pz, P4) is reported.

Note: When comparisons are made between studies using different EEG systems, filter settings can affect the P300 amplitudes. This analysis of the simple-concussion group data, for example, includes DC-drift settings not included in the previous study [32]. While these new filters had no effect on the trends, they did cause an overall 9% decrease in the P300 voltages reported here versus those previously published.

### 2.4.2 Spectra

Spectral analysis characterizes the frequency composition of EEG [51, 52] and can be used to identify peak frequency changes after trauma. In order to streamline testing times, and to be consistent with the clinical nature of this study, the alpha peak frequency and power spectra were extracted from the background eyes-closed EEG acquired during the auditory P300. Previous research has demonstrated that there are no significant differences between extracting alpha magnitudes and frequencies during an eyes-closed auditory P300 compared to an eyes-closed resting protocol [53]. Moreover, it is reasonable to hypothesize that the eyes-closed resting protocol may not be as stable due to lack of control of the state of the patient (e.g., drowsiness, concentration), while the P300 protocol requires a constant cognitive engagement. Furthermore, this study was consistent in that all groups used this same method.

In order to investigate spectral differences, the ratio of frontal to occipital alpha microvolt amplitudes and the occipital peak frequency was calculated. The frontal amplitudes represented the sum of the magnitudes at each of the alpha band frequencies (8–12 Hz) averaged across the five frontal locations: FP1, F3, FP2, F4 and Fz. This is equivalent to computing the sum of the square root of the power at each of the alpha band frequencies averaged across the same five frontal locations. Occipital alpha amplitude and peak frequencies were averaged over the occipital channels, O1 on the left and O2 on the right. Where the peak was not clear, no frequency was reported.

### 2.4.3 Connectivity

Alpha band coherence was extracted using standard methods described elsewhere [54]. As with the spectral analysis described above, eyes-closed coherence measures were extracted during the same eyes-closed P300 protocol. There are many methods of presenting coherence, but with N x (N-1)/2 = 171 pairs to choose from, care must be made to not overfit the data.

One method adopted here was a simple procedure in which the number of connections that were stronger than an age-matched reference was calculated by counting the number of connections larger than two standard deviations (2*σ*). Taken as the control, the pre-injury baseline group averaged three connections above 2 *σ* out of the 171, by definition. If trauma leads to more connections, particularly long-distance ones, then this method will allow for quantification of pre-post injury changes.

Calculated coherence metrics included frontal coherence (average of frontal scalp sites, FP1 to all F sites, F3 to all remaining F sites, etc.); occipital/parietal (average of all O and P scalp sites, O1 to P3, Pz, P4, T5, T6, and O2; P3 to the remaining sites; etc.). In order to test the hypothesis that trauma increases long-path connectivity, occipital-frontal connectivity (average of O1 to all F sites and O2 to all F sites) was calculated.

It should be noted that coherence, like many other EEG metrics, is susceptible to artifact, and the raw EEG must be visually inspected to ensure that artifact doesn’t affect long or short distance coherences. Alpha band coherence was used because it is less susceptible to motion artifacts and/or muscle tension than the other bands and because alpha often accounts for the greatest amount of power in the EEG band and thus the most robust coherence estimates.

Finally, it should also be noted that, while there are MRI studies linking EEG coherence to connectivity, [25, 54] coherence is not always a direct measure of connectivity. For example, the measured coherence between two cortical sites could result from the EEG activity at both sites being simultaneously driven by one or more additional cortical locations. In addition, the coherence measured between two locations can be a combination of active and passive (volume) conduction. While the use of both real and imaginary components of coherency have been proposed to distinguish between active and volume conduction, more work is needed in this area [55]. However, as mentioned above, since the focus of this study was on characterizing differences between baseline and post-injury, it was not necessary to distinguish between the real and imaginary components of the coherency.

## 2.5 Statistics

The mean of a given injury group (i.e., acute concussion, return-to-play, or PPCS) was compared to the pre-contact baseline groups using unpaired two-sample *t*-tests (because comparisons were made to baseline for all groups, multivariate tests were not used). A two-tailed *t*-test was used for all parameters except for long-path coherence because an increase was predicted and for the occipital alpha frequency at acute, return, and PPCS because a decrease was predicted. In response to a concern over the lack of reproducibility of certain medical studies, the *p* value cutoff was set to 0.05 with a minimum meaningful effect size of Cohen’s D > 0.50 [56]. On a final note, while the Gaussian nature of EEG can be debated, the metrics used here (P300 V, alpha magnitude and frequency, and coherence values) were sufficiently Gaussian for the simple-concussion group. The extent to which this held for the PPCS group was unclear, and with further exploration of phenotypes (as discussed below), different trajectories for each metric may be discovered. This was beyond the scope of this study.

## 3 Results

**Fig. 2:**
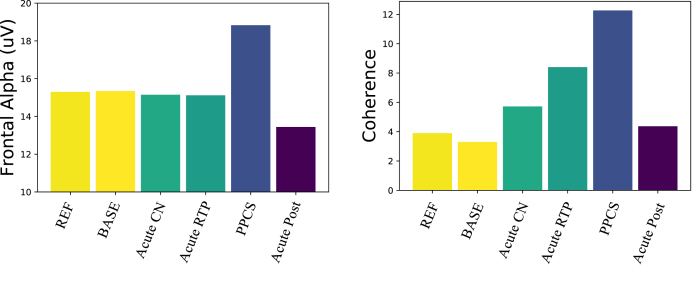
Electrophysical trajectories after concussion. (Left) Frontal alpha amplitude: only the PPCS group is significantly different from reference and pre-injury. (Right) Number of coherence connections in the alpha band above 2 *σ*: connectivity trends toward increased coherence from pre-injury baseline returning to expectation in the post-acute groups.

Table 2 compares the different stages in time for the metrics previously discussed: P300; the ratio of frontal-to-OP magnitude; occipital peak frequency; and the connectivity of the EEG signal. Here a clear pattern emerged. Both simple and complex concussion were marked by lingering P300 amplitude deficits (compared to both pre-injury baseline and baseline reference of non-injured players), but complex concussion had unique P300, spectral, and coherence features: significantly reduced occipital-parietal amplitude and frequency accompanied by increased frontal alpha amplitude; and increased interhemispheric connectivity. Note that no changes from the acute group’s baseline measures were found for any stage of recovery except for P300 amplitude and possibly an increased connectivity at return to play, as seen in Fig. 2.

**Table 2:** EEG and ERP metrics for various stages of concussion and recovery. Bold = significant difference from baseline. (OP = Occipital-Parietal as defined in the text)

Metric	Simple Concussion				Complex
*Pre-Injury *	*Acute *	*Acute RTP*	*Acute *	*PPCS*
*Baseline*	*Concussion*	*Post-Season*
***ERP***
P300 Voltage (SD)	17(5)uV	**10(4)uV**	**12(4)uV**	15(5)uV	**12(6)uV**
*Difference from Baseline P-Value (Cohen*’*s D)*	–	< ** 0.001(1.4)**	**0.001(1.1)**	*0.15*	<**0.001(0.8)**
P300 Latency (SD)	310(37)ms	308(48)ms	304(39)ms	318(45)ms	**278(25)ms**
*P-Value (Cohen*’*s D)*	–	*0.93*	*0.76*	*0.33*	< **0.001(0.9)**
**Spectral**
Frontal α Amplitude (SD)	15(5)uV	15(5)uV	15(4)uV	13(3)uV	**19(7)**
*P-Value (Cohen*’*s D)*	–	*0.87*	*0.84*	*0.11*	**0.02** *(0.5)*
OP α Amplitude (SD)	25(9)uV	23(9)uV	23(8)uV	**20(7)uV**	21(12)uV
*P-Value (Cohen*’*s D)*	–	*0.21*	*0.15*	**0.03(0.6)**	**.05** *(0.4)*
Frontal/OP α Amplitude (SD)	0.6(0.1)	0.7(0.2)	0.7(0.2)	0.7(0.2)	**1.1(0.6)**
*P-Value (Cohen*’*s D)*	–	*0.13*	*0.14*	*0.15*	< **0.001(1.0)**
Occipital Peak Frequency (SD)	10.2(0.7)Hz	10.2(0.9)Hz	10.2(0.6)Hz	10.5(0.8)Hz	**9.7(1.2)Hz**
*P-Value (Cohen*’*s D)*	–	*0.41*	*0.42*	*0.15*	**0.01(0.6)**
***Alpha-Band Connectivity***
Frontal Coherence (SD)	0.73(0.07)	0.69(0.12)	0.69(0.13)	**0.64(0.17)**	0.73(0.11)
*P-Value (Cohen*’*s D)*	–	*0.15*	*0.13*	**0.02(0.7)**	*0.83*
OP Coherence (SD)	0.50(0.07)	0.49(0.10)	0.51(0.09)	0.53(0.07)	**0.40(0.12)**
*P-Value (Cohen*’*s D)*	–	*0.68*	*0.71*	*0.14*	**<0.001(1.1)**
Occipital-Frontal Coherence (SD)	0.10(0.07)	0.12(0.06)	0.12(0.10)	0.09(0.07)	**0.21(0.15)**
*P-Value (Cohen*’*s D)*	–	*0.50*	*0.53*	*0.90*	**0.001(0.9)**
# of Coherence Connections > 2*σ*	3	5	**8**	4	**12**
	*0.10*	**0.03(0.5)**	*0.31*	**<0.001(1.1)**

## 3.1 ERP changes

These data showed a significant decline in P300 voltage after a concussive event, remaining in decline at return to play, and a clear tendency to return to baseline values for the acute concussion group, as seen in the acute post-season and next season. The PPCS group also showed a significant reduction in this ERP response amplitude compared to the pre-injured group. However, it appeared that the processing speed in the PPCS group may have been increased (decreased latency). Because the P300 latency becomes less reliable with lower amplitude, the latency pattern needs to be further investigated. Ongoing studies are aimed at following PPCS trajectories through recovery as well as subtyping the PPCS group with reduced P300 amplitudes based on symptom profiles [57].

## 3.2 Spectral changes

The alpha amplitude in the PPCS group trended toward reduction in occipital regions accompanied by a frontal increase; see Fig. 2 (left) and Fig. 1 (top). These trends were not typically seen in any other group. The PPCS group was also marked by a reduction in occipital peak frequency, also not seen in the other groups.

## 3.3 Changes in coherence

With respect to coherence changes in general, Fig. 2 (right) shows an increase in coherence in the alpha band for the PPCS group, with respect to coherence changes in the pre-injury and reference groups. As noticed in Fig, 2, the latter groups show 3-4 large connections (>2 *σ*) respectively, and the number of connections increases until it peaks at PPCS with 12 large connections, returning to expectation of 3-4 in the post-acute groups. As illustrated in Fig. 1 (bottom) and summarized in Table 2, the PPCS group uniquely demonstrated an increase in long interhemispheric coherences between occipital and frontal, though with moderate effect size, accompanied by no change in frontal connectivity and decreased occipital-parietal connectivity. Note that, in Table 2, the acute group’s pre-injury baseline occipital-frontal coherence was reduced from the reference group, but no change in this metric was seen in stages of recovery. As discussed, long-path coherence increases might indicate a spontaneous reorganization of the brain identified by an increase in functional brain connectivity during the healing process.

## 4 Case studies

To illustrate how these trends manifest clinically, an acute concussion case study 48 hours after an event and at return to play is presented. In addition, a case study involving a PPCS patient while symptomatic and after recovery and a case study involving a PPCS patient who had yet to recover are discussed.

### 4.1 Case study 1: Acute trajectories from baseline

**Fig. 3:**
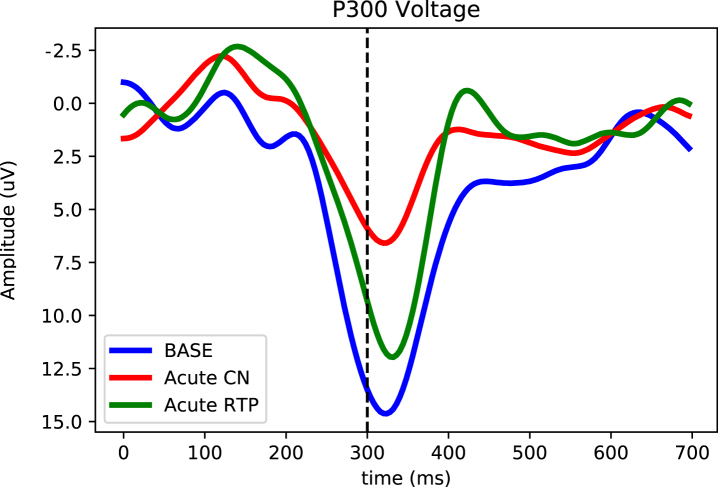
Longitudinal P300 magnitude tracking of an acute concussion at baseline (blue), 24–48 hours after the concussive event (green) and at return to play (red). Here the P300 voltage (averaged over the five central-parietal locations) is plotted as a function of time after delivery of the odd-ball stimulus. The strong downward (positive) peak at ∼300 ms in session 2 at symptom resolution is the P300. Notice that the P300 decreases after the concussion and increases significantly when subject cleared for return to play.

**Fig. 4:**
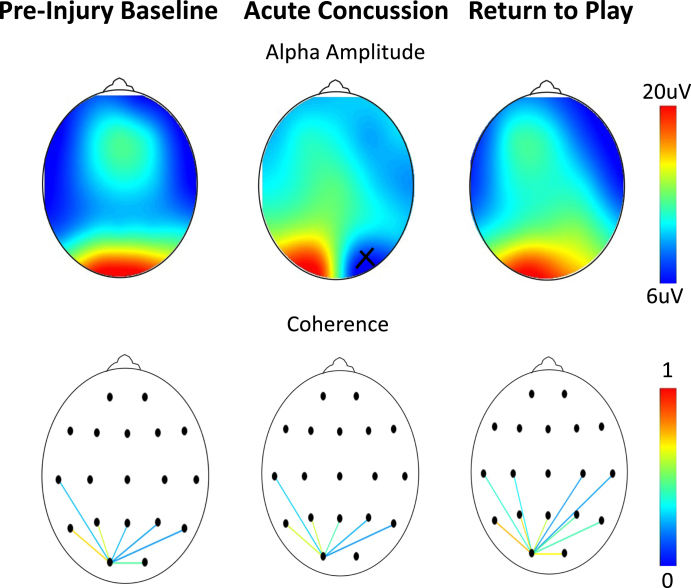
Assessments as a function of scalp location on an athlete at baseline, 48 hours after a concussion, and after symptom resolution one month later. (Top) Alpha amplitude (*x* = bad channel). (Bottom) Occipital connectivity (from O1 electrode, display threshold = 0.2). As per Table 2, no differences are seen in either the frequency or coherence.

The first case represents an athlete measured pre-injury and who then experienced a concussion two months later (Figs. 3-4). The first post-injury measure was obtained 48 hours after the event while the athlete was still very symptomatic. The next measurement was taken one month after the event when symptoms had resolved, and the player was cleared to begin return-to-play protocols. The electrophysiological trajectory for this athlete followed the trends of Table 2, in which the P300 changes after the event resolve at return to play (Fig. 3) although no other changes in the spectra or coherence were seen (Fig. 4).

### 4.2 Case study 2: PPCS with symptom resolution

The second case study involved a PPCS patient who was tested 2.5 months after the concussive event with moderate symptoms and a total SCAT5 symptom score of 64/123. The patient had abnormalities on oculomotor testing, vestibular testing, exertion tests, and VOMS screening. On the initial test, the patient underwent King Devick testing and Near Point Convergence (NPC) and scored in low/mid-level ranking. By the second test two months after the initial scan, he had near complete symptom resolution accompanied by a SCAT5 score of 2/126 and improved visual, cognitive, oculomotor, vestibular and exertion testing. King Devick scores and NPC were normal. Exertional tolerance had increased with no symptoms during exercise testing. Overall, there were very significant clinical changes in both subjective and objective functional measures between the two test/visit dates.

**Fig. 5:**
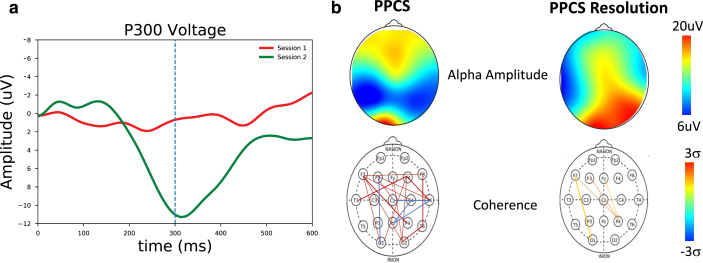
a) P300 measures for a PPCS patient (red) and at symptom resolution (green). Here, a strong P300 peak near ∼300 ms can be seen at symptom resolution but not in the first symptomatic PPCS session. b) Spectral and coherence measures for a PPCS patient and at recovery. (Top) Alpha amplitude as a function of scalp location. Significant changes are seen at recovery. (Bottom) Number of connections > 2 sigma. Significant long-path connections are seen at PPCS, as expected in accordance with Table 2, with normalization at recovery.

The electrophysiological trajectory for this patient, shown in Fig. 5, followed the trends of Table 2: P300 declines accompanied by alpha shifts and increased long-path connectivity all resolved to normal with symptom resolution.

### 4.3 Case study 3: PPCS with symptoms yet to resolve

The third case study involved a PPCS patient who was tested three months after the concussive event with mostly mild symptoms and a total SCAT5 symptom score of 20/132. The patient reported cognitive and visual symptoms along with trouble getting through work due to persistent cognitive fatigue. Mild abnormalities were evident upon oculomotor testing, vestibular testing, exertion tests, and VOM screening. Persistent symptoms were reported at the second test two months after the initial scan, with a SCAT5 score of 32/132. Symptoms still focused mostly on cognitive fatigue and visual profiles. Symptoms were persistent, and there were many significant clinical changes between the two test/visit dates.

**Fig. 6:**
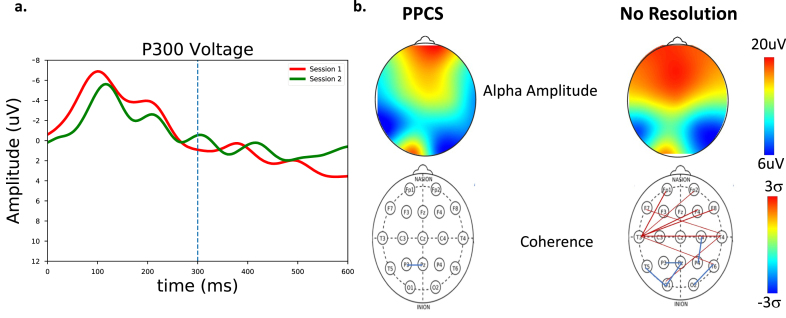
a) P300 measures for a patient with PPCS symptoms (session 1, red) and with continued symptoms (session 2, green). No clear P300 response emerges for this patient. b) Spectral and coherence measures for a PPCS patient and at follow up with no recovery. (Top) Alpha amplitude as a function of scalp location. (Bottom) Number of connections > 2 sigma. Significant long-path connections with reduced O-P connectivity are seen in the second PPCS session.

For this patient, the trajectory shown in Fig. 6 followed an interesting trend in which, with no symptom resolution, the P300 remained in decline, the frontal alpha shifts increased, and the increased long-path connectivity emerged on the second scan, raising interesting questions about the timing of connectivity changes in the healing process.

The differences between the first two case studies are compelling. Assessment of the simple concussion athlete revealed low P300 brain performance 48 hours after the event while the remaining metrics were unchanged from baseline. Here, the P300 resolution coincided with symptom resolution when the athlete began return-to-play protocols one month later. For the PPCS patient whose symptoms resolved, the first (symptomatic) scan produced spectra, P300, and coherences consistent with the low brain performance of prolonged symptomatology in the PPCS group, but the second scan looked very similar to the resolved simple concussion, perhaps a behavior representative of a restabilized brain. This normalization of spectra, P300, and coherence could therefore represent important steps in the PPCS healing trajectory.

The difference between the PPCS case studies (case studies 2 and 3) is also compelling. While the first patient seemed to stabilize, the second patient produced patterns consistent with the low brain performance seen with the lack of symptom reduction. In particular, the second session of the unresolved PPCS patient, illustrated in Fig. 6, looked very similar to the first session of the symptomatic PPCS session in both the spectral, P300, and coherence values. As in the resolved case, this second unresolved scan could represent a step in the healing trajectory.

## 5 Discussion

The goal of this study was to identify changes of brain functioning over time after a concussive event. Therefore, patterns of changes from baseline after acute concussion, during return to play, at the end of season, and for the prolonged PPCS group who had yet to return, were investigated by longitudinally tracking and monitoring changes in brains organization and performance over time as reflected in EEG changes. EEG and ERP markers at baseline and different stages of the healing process were compared and used as reference for more complex cases of prolonged post-concussion patients.

In the acute group, the lowered ERPs persisted at the time of initiating the return-to-play protocol (on average 10 days after event); this suggests that there may have been persistent impairments despite a presumed symptom-free athlete. Furthermore, although no changes in coherence were seen at the acute phase, an increase in coherence from the return to play through chronic stages was observed, suggestive of subacute or chronic remodeling phenomena as patients with more significant injuries re-established neural networks due to mechanisms of neuroplasticity.

While the persistent decrease in P300 amplitude for the PPCS population may reflect cognitive fatigue associated with these groups and/or may be due to a compensatory increase in norepinephrine output, this might also account for the increased alpha magnitude in the frontal regions and for the increase in coherence. Interestingly, these changes seemed to revert to baseline as the PPCS symptoms improved.

The lack of observed alpha reduction in the acute phase, in both frequency and amplitude, may suggest that the spectral changes in previously cited studies were on more complex traumas than the simple sports concussion. Studies on the PPCS population are ongoing in an effort to track symptom resolution and investigate symptomatic subtypes.

Perhaps the most striking result, however, was a significant increase in frontal alpha magnitude and frontal/occipital ration that was unique to the PPCS group. The relationship of this, and previous findings of reduced alpha power, to patient symptomatology and prognosis continues to be an interesting debate [15].

### 5.1 Implications

Analysis of the PPCS group revealed unique patterns beyond P300 amplitude suppression and elevated coherence, which included spectral shifts of alpha amplitude toward the front and reduced occipital-parietal alpha frequency. These trends may show significant ‘early’ patterns that predict PPCS or longer, more complex recoveries. These findings may also prove helpful in revealing persistent primary neurophysiologic brain dysfunction typically seen in the acute stages resulting from psychosocial dysfunction, which is common in the PPCS population. Presumably, patients with predominantly psychosocial symptoms or who are in pursuit of secondary gain would not show the NP patterns above that are consistent with persistent primary brain injury.

The combination of these findings suggests two important conclusions. First, primary neurophysiologic deficits appear to remain in patients with patterns (frontal alpha, coherence) that suggest an attempt at a healing process over time. Specifically, patients with PPCS may experience symptoms for months or even years, and thus data on where they may be in the course of recovery could be crucial to the practitioner and patient. More research will have to be done to substantiate these findings, but at this time, they do represent very interesting trends. Second, the results suggest that the combined use of EEG and ERP is a potentially useful tool to objectively assess brain injury diagnosis, recovery course, and prognosis. The use of ERP can be a helpful tool to aid in the subjective assessment of patients with prolonged recoveries accompanied by ongoing complaints of persistent concussive symptoms. EEG can also help in monitoring the healing process with what are believed to be important markers for PPCS, which can also support decision making in the multi-disciplinary care settings often required by the PPCS group. With ongoing evidence of neurophysiologic dysfunction such as frontal alpha, coherence, P300 amplitude, and latency, ERPs can be a potentially critically useful tool identifying patients at risk of developing prolonged symptoms and also be used to subtype concussions in order to direct early management with targeted treatments.

### 5.2 Limitations

Potential shortcomings of the study include the heterogenous composition of the study population. Since patient data were collected during the course of routine examinations, the mechanisms of the injuries were varied, as were their medication profiles and histories of previous concussion. Also, the percentages of males versus females varied between the studies. While no gender differences were seen, there were not enough samples to make any gender-related comparisons and to eliminate this potential variable. Other considerations, not unrelated, include the potential that the metrics measured here bifurcate into non-Gaussian distributions in the PPCS population. For example, does the large frontal-alpha population represent a particular deviation from the rest of the PPCS population? As of the writing of this paper, the data sample was not large enough to stratify these populations. However, as more data are collected, differences between PPCS phenotypes and past histories should be explored. Although computerized cognitive testing was done in the entire sample with potential cognitive abnormalities being an indication for performing P300 evoked potential testing, correlations to specific cognitive markers or cognitive scores were not analyzed. In the same vein, correlations from formal neuropsychiatric testing results, which most of the cohort had completed, were not analyzed in this study but certainly do mark future directions.

Finally, the acute group had baseline tests allowing real trajectories to be followed. The PPCS group did not. As more data are collected, true baseline-to-acute-to-PPCS trajectories can be studied to better understand acute-to-prolonged transitions.

## 6 Conclusions

These data suggest that the ERP deficits of simple concussion may persist in more prolonged stages, which may be reflective of cognitive fatigue as the most commonly reported symptom in both the acute and PPCS cohorts. There are, however, EEG spectral and coherence measures unique to the PPCS group as well. Early recognition of these unique PPCS markers may help clinicians identify patients at higher risk for PPCS and implement evidence based ‘early intervention,’ thus reducing and/or preventing PPCS-related morbidity. ERP and EEG differences may also help in the future identification of phenotype trajectories and the implementation of targeted treatments for phenotypes (i.e., vestibular rehabilitation for vestibular phenotype vs. vision therapy for visual phenotype). These metrics are readily accessible to clinicians and may provide useful tracking parameters to track the time course of injury.
